# Influence of Refrigerated Storage on Water Status, Protein Oxidation, Microstructure, and Physicochemical Qualities of Atlantic Mackerel (*Scomber scombrus*)

**DOI:** 10.3390/foods10020214

**Published:** 2021-01-21

**Authors:** Rong Lin, Shasha Cheng, Siqi Wang, Mingqian Tan, Beiwei Zhu

**Affiliations:** 1 National Engineering Research Center of Seafood, School of Food Science and Technology, Dalian Polytechnic University, Dalian 116034, China; 18308320017021@xy.dlpu.edu.cn (R.L.); 17308320017012@xy.dlpu.edu.cn (S.W.); mqtan@dlpu.edu.cn (M.T.); zhubw@dlpu.edu.cn (B.Z.); 2 Collaborative Innovation Center of Seafood Deep Processing, Dalian 116034, China; 3 National Engineering Research Center of Seafood, Dalian 116034, China

**Keywords:** water status and distribution, microstructure, secondary structure of protein, Atlantic mackerel

## Abstract

Moisture migration, protein oxidation, microstructure, and the physicochemical qualities of Atlantic mackerel during storage at 4 °C and 0 °C were explored in this study. Three proton components were observed in mackerel muscle using low-field nuclear magnetic resonance relaxation, which were characterized as bound water, immobilized water, and lipid. The relaxation peak of immobilized water shifted to a shorter relaxation time and its intensity decreased with the proceeding of the storage process. *T*_1_ and *T*_2_ weighted images obtained by magnetic resonance imaging showed a slightly continuous decrease in the intensity of water. The significant decrease in sulfhydryl (SH) content and the increase in carbonyl group (CP) content, disulfide bond content, and hydrophobicity revealed the oxidation of protein during storage. The contents of α-helixes in proteins decreased while that of random coils increased during storage, which suggested changes in the secondary structure of mackerel protein. The storage process also caused the contraction and fracture of myofibrils, and the granulation of endolysin protein. In addition, the drip loss, total volatile basic nitrogen (TVB-N), thiobarbituric acid-reactive substances (TBARS) value, and b* value increased significantly with the storage time.

## 1. Introduction

Atlantic mackerel (*Scomber scombrus*) is an abundant pelagic marine fish with various nutrients such as polyunsaturated fatty acids, high quality protein, and vitamin D, which is consumed by consumers worldwide [1]. The total protein content in white muscle of Atlantic mackerel is between 18.5% and 20.8% [2]. The lipid content of Atlantic mackerel can be as high as 29.7% at the optimal fishing location and season [3]. However, Atlantic mackerel easily decomposes within a short period after fishing because of its high water activity, high enzymatic activity, high protein content, and high lipid content, which leads to a series of quality deterioration such as drip loss, lipid oxidation, protein decomposition, microbial growth, and pH change [4,5,6]. In recent years, low-temperature preservation has become an effective method to maintain the quality of aquatic products by reducing the activities of endogenous enzymes and spoilage microorganisms [7].

The total moisture content of the Atlantic mackerel muscle is up to 70% [2]. The water status and distribution are critical factors affecting the physical and chemical qualities of fish and fish products including sensory quality, water holding capacity (WHC), drip loss, and texture [8]. Low-field nuclear magnetic resonance (LF-NMR) and magnetic resonance imaging (MRI) have successfully realized the monitoring of water states and distribution in food during storage and processing as non-destructive and rapid technologies [9]. LF-NMR is commonly used to evaluate the proton relaxation behavior including longitudinal (*T*_1_) relaxation and transverse (*T*_2_) relaxation, in which *T*_2_ relaxation could provide information about the interactions between water molecules and macromolecules (proteins) in muscle tissues [10]. The water within aquatic products are usually characterized as three populations, which are ascribed to bound water closely associated with macromolecules, immovable water located within organized protein structures and free water within the extra-myofibrillar [8]. Meanwhile, MRI provides additional spatial information about nuclear spins in the sample, which can visualize the spatial distribution of water. Andersen et al. [11] observed the close correlation between the full NMR-signal decays and WHC of cod subjected to various storages of frozen and/or chilled conditions up to 24 months. Sánchez-Alonso et al. observed three populations of water in hake using LF-NMR relaxometry and investigated the effects of different freezing mode and frozen conditions on the water status and distribution [12,13]. Obvious decreases in the relaxation time and abundance of immobilized water were observed in hake during frozen storage, and the obtained LF-NMR parameters could realize the prediction of frozen storage time and quality changes [12,13]. Sánchez-Valencia et al. also found that the frozen storage could cause decreases in relaxation time and abundance of immobilized water in hake, and the higher frozen temperature accelerated the decrease [14]. The increase in relaxation time of immobilized and free water was observed in channel catfish fillets during frozen storage, accompanied by the decrease in the abundance of immobilized water and the increase in the abundance of free water [15]. Ultrasound-assisted immersion freezing could reduce the increase in the freedom of immobilized and free water in common carp during frozen storage at −18 °C [16]. Three water components were observed in grass carp, in which the immobilized water was identified as the major water component and became more mobile during super-chilled storage with the addition of salt and sugar [17]. Sun et al. observed obvious moisture migration between bound, immobilized, and free water in mandarin fish in the early stage of 4 °C storage due to the change in protein–water interaction [18]. Wang et al. investigated the changes of water distribution in salmon [19], yellow tuna, and bigeye tuna [20] during storage at 0 and 4 °C using LF-NMR and MRI and found that the trapped water decreased while free water increased with the prolongation of storage time and the relaxation parameters had significant correlations with the protein and quality changes of fish muscle. Salting methods and modified atmosphere packaging could affect the water dynamic parameters of the muscle and muscle structure during superchilled storage [21]. Previous research found three proton components in Atlantic mackerel, and the catching season, frozen storage temperature, and duration affected the relaxation time and abundance of protons [22]. However, there is limited information about the moisture migration of Atlantic mackerel during refrigerating storage.

The quality deterioration of fish during preservation is also associated with protein degradation, protein aggregation, or protein crosslinking, which will lead to changes in the structure and conformation of the protein [23]. Previous studies have found that the protein oxidation occurred in fish under different storage conditions, which affected the elasticity of the protein gel, the total sulfhydryl (SH) and carbonyl group (CP) content of protein as well as the solubility and surface hydrophobicity of the protein [24,25]. Refrigeration storage at 4 °C caused the decrease in the contents of salt soluble protein, water soluble protein, and total protein of hybrid catfish fillets [26]. The softening of fish fillets during cold storage was attributed to the collagen solubilization and myofibrillar protein hydrolysis [27]. Detachment between myofibers and myocommata was concomitant with the loss of dystrophin, which corresponded to the reduction in flesh hardness of sea beam during ice storage [28]. Protein degradation was promoted by protein oxidation, which increased protein surface hydrophobicity and changed the secondary structure during refrigerated storage [29].

**Scheme 1:**
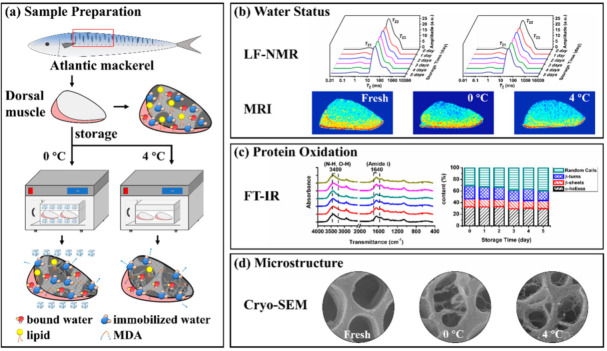
Schematic diagram of this paper. (**a**) Sample preparation, (**b**) Water status, (**c**) Protein oxidation, (**d**) Microstructure. (Low-field nuclear magnetic resonance (LF-NMR), magnetic resonance imaging (MRI), fourier transform infrared spectra (FT-IR), scanning electron microscopy (SEM) and malondialdehyde (MDA)).

The aim of this study was to explore the changes in moisture migration, protein oxidation, microstructure, and the physicochemical quality of Atlantic mackerel in refrigerated conditions with and without ice (Scheme 1). The moisture distribution and migration of the Atlantic mackerel during the refrigeration process were monitored by LF-NMR and MRI. Changes in the secondary structure of protein were determined by Fourier transform infrared spectra (FTIR) and CP content, total SH content, disulfide bond content, and surface hydrophobicity were analyzed to evaluate the protein oxidation of mackerel muscle during refrigerated storage. The microstructure of mackerel muscle was observed using cryo-scanning electron microscopy. In addition, physicochemical properties including pH, total volatile basic nitrogen (TVB-N), thiobarbituric acid-reactive substances (TBARS), color, and other indicators were also analyzed to evaluate the quality changes.

## 2. Materials and Methods

### 2.1. Sample Preparation

Vacuum-packed Atlantic mackerel (*Scomber scombrus*) fillets were purchased from Qingdao Yihexing Foods Co. Ltd. (Qingdao, Shandong province, China) and expressed to the laboratory under freezing conditions. Average weight of the fillets was 150 ± 10 g. Before the experiment, the frozen fillets were thawed under flowing water for half an hour. The dorsal muscles of Atlantic mackerel with 3.0 cm width and 2.0 cm thickness were cut into 3.0 cm length pieces. The mackerel pieces were placed in polythene bags, and randomly divided into two groups, which were stored in a refrigerator without ice (4 °C) and with ice (0 °C, ice replaced every day). The thawed fillets were defined as the sample stored for 0 day in this experiment.

### 2.2. Low-Field Nuclear Magnetic Resonance (LF-NMR) and Magnetic Resonance Imaging (MRI) Measurements

*T*_2_ transverse relaxation data were measured by a MesoQMR23-060H NMR analyzer (Suzhou Niumag Analytical Instrument Co., Suzhou, China). The permanent magnet was kept at 32 °C with a 0.5 T magnetic field strength. A Carr–Purcell–Meiboom–Gill (CPMG) sequence with parameters including 90° pulses, 180° pulses, and π-value of 21.00 μs, 42.00 μs, and 200 μs was used to collect the relaxation decay data. After removing the surface water, the sample was placed in the 60-mm-diameter tube NMR probe and measured under 10,000 echoes with four scan repetitions. The inversion of the CPMG decay data was performed using MultiExp Inv analysis software (Suzhou Niumag Analytical Instrument Co., Suzhou, China) to obtain the *T*_2_ distributed curve as well as the relaxation parameters of different proton components.

MRI experiments were performed by the same MesoQMR23-060H NMR analyzer with spin echo (SE) imaging sequence. The scanning parameters of matrix size, field of view, and slice width were 256 mm × 196 mm, 100 mm × 100 mm, and 3 mm, respectively. The echo time (*T*_E_) and repetition time (*T*_R_) of the *T_1_* weighted image were 40 ms and 660 ms, respectively, while that of the *T_2_* weighted image were 40 ms and 3300 ms. The raw MRI images were processed using OsiriXLite software (v7.0.4, Pixmeo, Geneva, Switzerland) to obtain pseudo-color images and the corresponding intensities.

### 2.3. Protein Oxidation

#### 2.3.1. Protein Extraction

Water- and salt-soluble proteins were extracted from the mackerel muscle according to Yang et al. [23]. First, 5 g of mackerel muscle was mixed with 20 mL of precooled 20 mmol/L Tris-maleate solution (50 mmol/L NaCl, pH 7.0). The mixture was homogenized for 45 s on ice and centrifuged for 10 min at 4 °C with a centrifugal force of 8000× *g*. The supernatant was used for the measurement of water-soluble sarcoplasmic protein. Afterward, 20 mL of precooled 20 mmol/L Tris-maleate solution (0.6 mol/L NaCl, pH 7.0) was added to the precipitate, and the mixture was homogenized for 45 s on ice. After 15 min centrifugation at 8000× *g* at 4 °C, the supernatant was collected as the salt-soluble myofibrillar protein solution for subsequent experiments. The contents of water- and salt-soluble protein in the supernatant were measured using the method of Bradford, and bovine serum albumin (BSA) was used as the standard.

#### 2.3.2. Analysis of Carbonyl Group (CP) Content, Total Sulfhydryl (SH) Content, Disulfide Bond Content, and Surface Hydrophobicity

According to the previous method of Ganhao et al. [30], 1 mL of 2.4-dinitrophenol hydrazine (DNPH, 10 mmol/L in 2 N HCl) was mixed with 1 mL of the salt-soluble protein solution, which was incubated in a 30 °C water bath for 1 h in darkness. Then, the protein in the mixture was precipitated by the addition of 1 mL of trichloroacetic acid. After 5 min centrifugation at 8000× *g*, the precipitate was washed three times using 1 mL ethanol–ethyl acetate (1:1, *v*/*v*) solution to remove the redundant DNPH. Finally, the precipitate was suspended in 3.5 mL guanidine hydrochloride (6 mol/L) and kept at 37 °C for 15 min. After 3 min centrifugation at 10,000× *g*, the absorbance of the supernatant was measured at 370 nm to calculate the content of carbonyl groups expressed as μmol of per gram of protein.

The total SH content was determined according to Shi’s method [25] with some modifications. First, 9 mL tris-HCl buffer (0.05 mol/L with 8 mol/L urea, 10 mmol/L ethylenediamine tetra acetic acid (EDTA, pH 6.8) was added to 1 mL myofibrillar protein solution, which was incubated at 4 °C for 1 h. Then, 4 mL of the mixed solution was sampled and mixed with 400 μL Ellman’s reagent (DTNB). After incubating for 25 min at 40 °C, the absorbance of the solution was measured with a wavelength of 412 nm, and the total SH content was calculated as μmol per gram protein with 13,600 M^−1^ cm^−1^ as the molar extinction coefficient.

The content of the disulfide bond was calculated by the total SH and free SH content. The free SH content was measured using the same method of total SH except for the tris-HCl buffer, which was substituted by an equal volume of buffer without urea (0.05 mol/L tris-HCl with 10 mM EDTA, pH 6.8). The disulfide bond was calculated with the following equation:

(1) cdisulfide bond=(ctotal SH−cfree SH)2

Changes of surface hydrophobicity were determined according to the methodology of Kobayashi et al. [31]. For this experiment, 8-Anilinonaphthalene-1-sulfonic acid (ANS, 8 mmol/L in 0.1 mol/L PBS buffer, pH 7.0) was used as the fluorescent probe. Myofibrillar protein solutions with gradient concentrations of 0.05, 0.10, 0.15, and 0.2 mg/mL were prepared by the dilution of stock solution. Then, 4 mL of the diluted myofibrillar solution was mixed with 10 μL ANS. The fluorescence intensity of the mixture was determined by an F-2700 Fluorescence Spectrophotometer (Hitachi, Tokyo, Japan) using a 390 nm excitation wavelength and 470 nm emission wavelength. S_0_-ANS was calculated based on the slope of the curve between fluorescence intensity and protein concentration.

#### 2.3.3. Fourier Transform Infrared (FTIR) Spectra

Freeze-dried myofibrillar protein was used for the FTIR analysis (Frontier, PerkinElmer, Norwalk, CT, USA). The sample was mixed with KBr (1:100), and the mixture was ground into a uniform powder and pressed into a transparent sheet. Under transmittance mode, all spectra were collected in the wavelength range of 4000 to 400 cm^−1^ with the resolution of 4 cm^−1^ at room temperature. Omnic software was used for basic spectrum analysis. The data of the amide I bands were fitted to a Gaussian curve by Peakfit 4.12 software (Seasolve Software, Inc. Framingham, LA, USA). The secondary structure contents of each component were calculated from the peak area.

### 2.4. Microstructure

Samples with different storage times were cut into 2.0 mm × 2.0 mm × 5.0 mm pieces for microstructural analysis using cryo-scanning electron microscopy (SU8010/PP3010T, Hitachi, Tokyo, Japan). The pieces were placed on a sample tray coated with carbon conductive adhesive and immersed into slush nitrogen (−210 °C) to cryo-fix. The sample tray was transferred into a vacuum cryo-preparation chamber, and fractured on a preparation stage at −140 °C to expose the internal structure. After sublimating at −90 °C for 10 min, the sample was sputtered for 60 s at a 10 mA current, and transferred into the scanning electron microscopy (SEM) chamber for observation. The accelerating voltage of the electron beam was set at 1.0 kV.

### 2.5. Physicochemical Parameters

#### 2.5.1. Drip Loss

The samples were taken out of the polythene bags and blotted with filter paper before weighing. Drip loss was calculated based on the weight difference of the mackerel samples before and after storage.

#### 2.5.2. pH Value

The mixture of mackerel muscle and distilled water in aa ratio of 1:5 (w/v) was homogenized, and the suspension was used for pH value measurement with a digital pH meter (S210 Seven compact, Mettler-Toledo Instrument Co., Ltd., Shanghai, China).

#### 2.5.3. Total Volatile Basic Nitrogen (TVB-N)

TVB-N was measured according to Conway’s micro-diffusion method of association of official analytical chemists (AOAC) [32]. Briefly, the mixture of 5 g of the homogenized mackerel muscle and 50 mL distilled water was placed in a conical flask at ambient temperature for 30 min and filtered. Then, 1 mL of 2% boric acid was added in the inner ring of the Conway dishes, while 1 mL of the filtrate and 1 mL of saturated K_2_CO_3_ solution were placed in the outer ring. Each dish was covered by a piece of glass and sealed with gum arabic. The filtrate and saturated K_2_CO_3_ solution in the outer ring were mixed quickly. The Conway dishes were kept at 37 °C for 2 h, in which the formed volatile basic compounds were absorbed by the boric acid in the inner ring. The boric acid absorption solution of each Conway dish was titrated by H_2_SO_4_ with a micro-burette until it turned from a green color to pink. The TVB-N value was expressed as mg/100 g fish muscle according to the consumption of H_2_SO_4_:

(2) $$\mathrm{TVB}-N\;\left(\mathrm{mg}/100\;g\right)=\frac{V_{H_{2}{\mathrm{SO}}_{4}}\times c_{H_{2}{\mathrm{SO}}_{4}}\times 14}{m_{\mathrm{sample}}}\times 50\times 100$$

where V_H2SO4_ an c_H2SO4_ are the volume and concentration of H_2_SO_4_ solotion, and m_sample_ is the mass of mackerel muscle.

#### 2.5.4. Thiobarbituric Acid-Reactive Substances (TBARS)

TBARS values were measured according to John et al. [33] to estimate the level of lipid oxidation of Atlantic mackerel. First, 5 mL mixed solution (15% trichloro-acetic acid, 0.375% thiobarbituric acid, and 0.25 N HCl) was added to 1 g minced fish. Then, the mixture was incubated for 20 min in boiling water bath and centrifuged for 15 min at 8000× *g* by a centrifuge (CF-RXII Hitachi centrifuge, Hitachi, Japan). The supernatant was used to measure the absorbance at 532 nm by a spectrophotometer (Infinite F200 PRO, Tecan, Mannedorf, Switzerland). The content of malondialdehyde (MDA) was calculated based on absorbance and converted to a TBARS value as follows: TBARS (mg/kg) = A_532 nm_ × 2.77.

### 2.6. Color Measurement

The surface color of mackerel were determined in CIE L* (lightness) a* (redness) b* (yellowness) by an USP1792 UltraScan PRO colorimeter (Hunter Associates Lab., Inc., Reston, VA, USA). The colorimeter was equipped with a 10° standard observer, a D65 light source, and an aperture with 0.19 inch diameter. Three samples for each storage condition were prepared for color analysis every day, and three different surface areas of each sample were selected to measure the color value.

### 2.7. Statistical Analysis

All experimental data were displayed as mean values ± standard deviation. A commercial SPSS 19.0 software (SPSS Inc., Chicago, IL, USA) was used for the significance analysis of experimental data with one-way ANOVA followed by Tukey’s test. Parts of the figures were plotted using Origin 8.5 software (OriginLab Corporation, Northampton, MA, USA).

## 3. Results

### 3.1. LF-NMR and MRI Analysis

**Figure 1:**
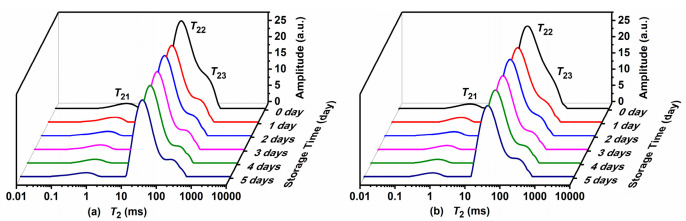
*T*_2_ curves of Atlantic mackerel during five days of storage at (**a**) 4 °C and (**b**) 0 °C.

The proton distribution and characteristics of Atlantic mackerel during storage at 4 °C and 0 °C were analyzed by LF-NMR proton transversal relaxation measurements, and *T*_2_ distributed curves are shown in Figure 1a,b, respectively. Three proton peaks labeled as *T*_21_, *T*_22_, and *T*_23_ with different relaxation times were observed in the Atlantic mackerel matrix. *T*_21_ with a relaxation time less than 10 ms was described as bound water closely attached on the polar groups of macromolecules. The strongest *T*_22_ peak was ascribed to immobilized water, which was entrapped within the myofibrillar network based on its relaxation time in the range of 10–100 ms. *T*_23_ with a relaxation time between 100–1000 ms appeared as a shoulder peak of *T*_22_, which was obviously different from the free water peak reported in other muscle food [8]. To identify the assignment of *T*_23_, the *T*_2_ relaxation distribution of fresh and freeze-dried Atlantic mackerel samples were compared, as shown in Figure S1. After the freeze-drying, the bound water *T*_21_ and the immobilized water *T*_22_ disappeared. Only one predominant proton peak with relaxation time around 100 ms was observed in the freeze-dried sample, which was ascribed to lipid based on the high lipid content of Atlantic mackerel. Therefore, the *T*_23_ peak of Atlantic mackerel sample was assigned to the protons of lipid.

**Figure 2:**
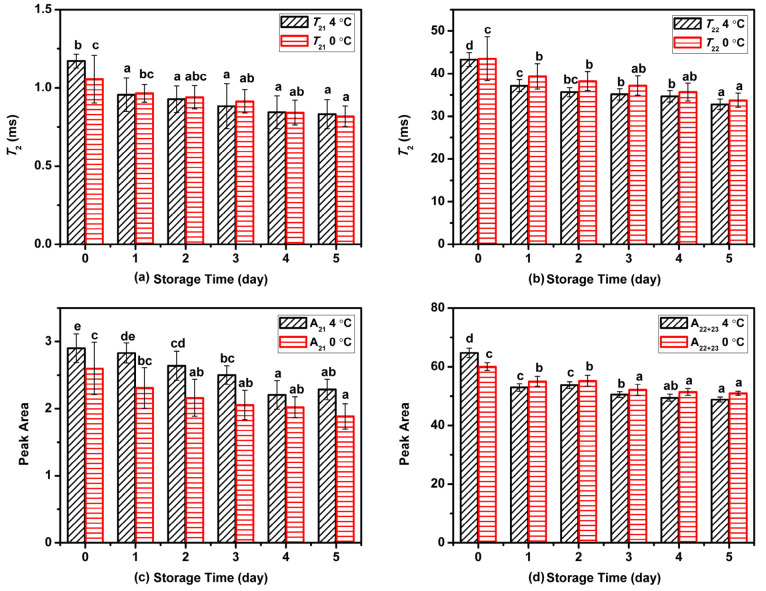
Variation in (**a**) *T*_21_, (**b**) *T*_22_, (**c**) A_21_, and (**d**) A_22_ of water components in Atlantic mackerel during storage at 4 °C and 0 °C for five days. Different letters a–d mean significant difference (*p* < 0.05).

The changes in relaxation parameters for Atlantic mackerel during two storage conditions are shown in Figure 2. With the proceeding of refrigeration storage at 4 and 0 °C, the relaxation peaks of *T*_21_ and *T*_22_ shifted to shorter relaxation time, suggesting the decrease in water mobility of Atlantic mackerel during storage. The decrease of *T*_22_ might be caused by the denaturation and aggregation of protein, which led to changes in chemical exchange between water and protein protons [13,20]. As shown in Figure 2c,d, the change in the peak area of A_21_ was nonsignificant, while A_22_ dropped rapidly on the first day, and then decreased slightly during subsequent storage. The status and distribution of water could influence other qualities such as WHC and textural properties.

**Figure 3:**
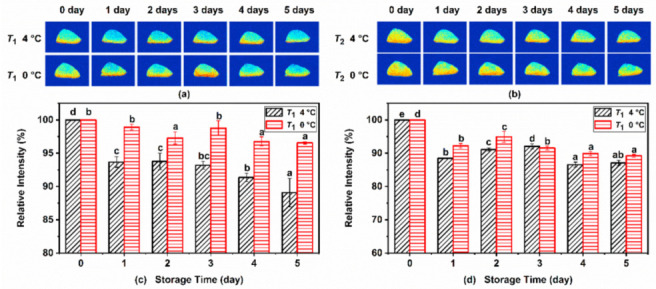
(**a**) *T*_1_ and (**b**) *T*_2_ weighted magnetic resonance imaging (MRI) images of Atlantic mackerel during storage at 4 °C and 0 °C, and the relative intensities of (**c**) *T*_1_ and (**d**) *T*_2_ images. Different letters a–e mean significant difference (*p* < 0.05).

Figure 3a,b shows the *T*_1_ and *T*_2_ weighted pseudo-color images of Atlantic mackerel under different refrigeration conditions, in which the red color represents high proton density and the blue color represents low proton density. Protons with low and high mobility are highlighted in the *T*_1_ and *T*_2_ images, respectively. As shown in the pseudo-color image, the signal intensity was mainly distributed in the skin and the dark meat near the skin. During storage of Atlantic mackerel, the MRI intensity was weakened at both 4 °C and 0 °C, suggesting that the water content of the fish decreased during refrigeration storage. The quantitative signal intensity of the *T*_1_ and *T*_2_ weighted images are shown in Figure 3c,d. Significant decreases in the intensities of the *T*_1_ and *T*_2_ images under two storage conditions with the storage time were observed, which was consistent with the decrease in the *T*_21_ and *T*_22_ + *T*_23_ peak area observed in *T*_2_ relaxation. The decrease in the signal intensity of *T*_1_ and *T*_2_ images demonstrated the decrease in the moisture content of mackerel muscle, which could be ascribed to the degradation and denaturation of proteins during refrigeration.

### 3.2. Protein Oxidation Analysis

**Figure 4:**
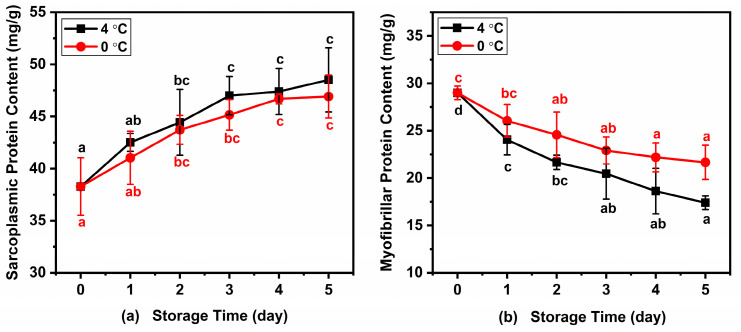
Variations in (**a**) sarcoplasmic protein and (**b**) myofibrillar protein of Atlantic mackerel during five days storage at 4 °C and 0 °C. Different letters a–d mean significant difference (*p* < 0.05).

The water-soluble protein in fish is mainly sarcoplasmic protein, while the salt-soluble protein is mainly myofibrillar protein. Changes in sarcoplasmic protein and myofibrillar protein of Atlantic mackerel during five days of storage at 4 °C and 0 °C are shown in Figure 4. The initial content of water-soluble protein in Atlantic mackerel stored for 0 days was 38.28 ± 2.76 mg/g, which increased to 48.52 ± 3.07 mg/g and 46.92 ± 2.06 mg/g after five days at 4 °C and 0 °C, respectively. Meanwhile, the content of myofibrillar protein in Atlantic mackerel before storage was 29.01 ± 0.72 mg/g, which decreased to 17.40 ± 0.72 mg/g and 21.67 ± 1.80 mg/g after five days at 4 °C and 0 °C, respectively. The decrease in salt-soluble protein content of Atlantic mackerel stored at 4 °C was more significant than that of 0 °C. The protein oxidation, leading to protein crosslinking and protein aggregation, could be explained by the decrease in salt-soluble protein extractability during storage [34]. The increase in the water-soluble protein content of Atlantic mackerel during storage corresponded to the decrease in salt-soluble protein content, which was in agreement with the study on bighead carp fillets under chilled storage [35].

**Figure 5:**
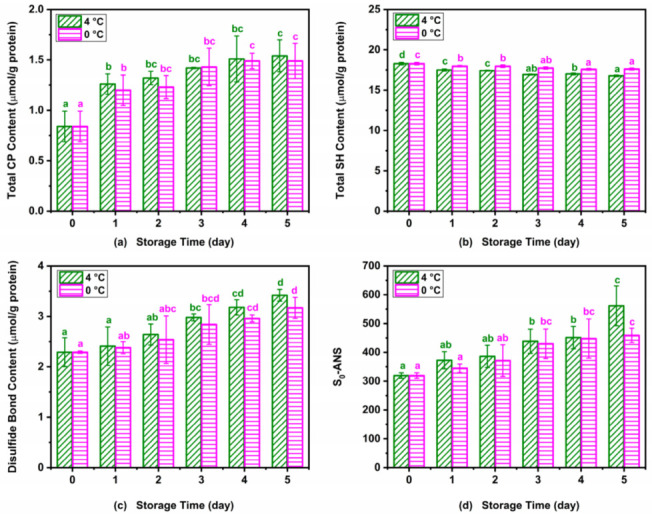
Variations in (**a**) carbonyl, (**b**) total sulfhydryl, (**c**) disulfide bond, and (**d**) surface hydrophobicity (S_0_-ANS) of Atlantic mackerel protein during five days of storage at 4 °C and 0 °C. Different letters a–d mean significant difference (*p* < 0.05).

Changes in CP content, sulfhydryl groups, and hydrophobicity are usually used to quantify the protein oxidation of muscle food [36]. Figure 5 shows the changes in CP content, sulfhydryl group content, disulfide bond content, and surface hydrophobicity of Atlantic mackerel during 4 °C and 0 °C storage. The carbonyl content of myofibrillar in Atlantic mackerel showed a significant increase from an initial value of 0.84 ± 0.15 μmol/g at 0 day to 1.54 ± 0.16 and 1.49 ± 0.18 μmol/g on the fifth day stored at 4 °C and 0 °C, as shown in Figure 5a. The metal-catalyzed oxidation, and reaction with lipid peroxidation products and the oxidation of the side chain or backbone of some amino acids could all produce carbonyl groups, which reduces the protein solubility [36]. Similar results were also observed in the minced mackerel during 4 °C storage, which suggested the relationship between the formation of protein carbonyl groups and lipid oxidation [37]. The total SH content showed a decrease from an initial value of 18.28 ± 0.15 µmol/g to 16.77 ± 0.09 and 17.63 ± 0.12 µmol/g protein at 4 °C and 0 °C, respectively (Figure 5b). Corresponding to the decrease in the total SH content, the disulfide bond content of myofibrillar protein from Atlantic mackerel increased significantly during the 4 °C and 0 °C storage (Figure 5c). The formation of disulfide bonds was probably ascribed to the oxidation of SH groups of the protein during the refrigeration storage [20]. The surface hydrophobicity of Atlantic mackerel protein significantly increased during storage at 4 °C and 0 °C (Figure 5d), which indicated the occurrence of protein denaturation. The increase in the surface hydrophobicity could be explained by the unfolding of the proteins during cold storage, which led to the exposure of hydrophobic aliphatic and aromatic amino acids [20]. The above results show that the storage at 0 °C could diminish the protein oxidation of Atlantic mackerel compared with storage at 4 °C.

### 3.3. Secondary Structure Change of Atlantic Mackerel Protein

**Figure 6:**
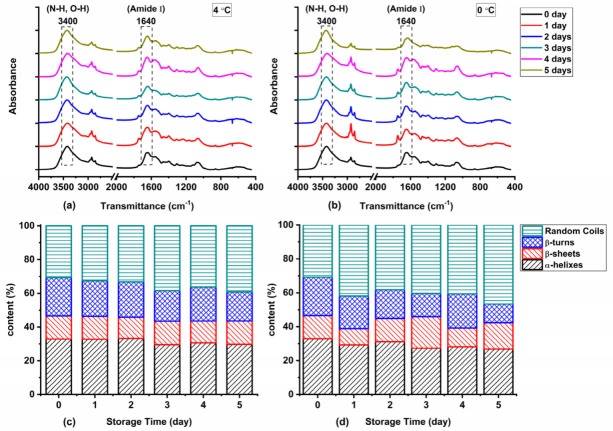
Fourier transform infrared (FTIR) spectra of Atlantic mackerel protein stored at (**a**) 4 °C and (**b**) 0 °C after different storage times; quantitative analysis of secondary structure changes of protein in Atlantic mackerel during storage at (**c**) 4 °C and (**d**) 0 °C.

The degradation and oxidation of proteins during storage generally lead to the destruction of secondary structure and spatial conformation of proteins. The FTIR spectra of protein extracted from Atlantic mackerel with different storage times under two storage conditions are shown in Figure 6a,b. The amide I band (1600~1700 cm^−1^) was the main characteristic absorption band of protein in the infrared region. The FTIR spectra of Atlantic mackerel stored at 4 °C and 0 °C both showed a strong absorption peak around 1640 cm^−1^, and the absorption intensity increased with the prolongation of storage time, which might be due to the changes in the conformation of the polypeptide chain. The percentages of α-helices, β-turns, β-sheets, and random coils of protein extracted from Atlantic mackerel under different storage conditions are shown in Figure 6c,d. Decrease in percentage of α-helix and increase in percentage of random coils were revealed by the quantitative analysis. The network structure was damaged due to the protein aggregation by the cross-linking of disulfide bonds and carbonyls [38]. The decrease in α-helices indicated the unfolding of protein induced by oxidation, which was consistent with the increase in hydrophobicity. The changes of β-turns and β-sheets were also ascribed to the oxidation of proteins, which was promoted by the free radicals produced by the lipid oxidation [20].

### 3.4. Microstructure Analysis

**Figure 7:**
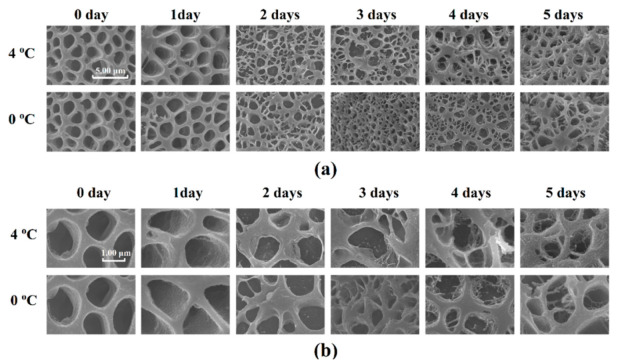
Microstructure of Atlantic mackerel after storage at 4 °C and 0 °C observed by cryo-scanning eletron microscopy (Cryo–SEM) with (**a**) 10 k× and (**b**) 30 k×.

Protein denaturation can be reflected in the microstructure. The qualitative changes in the microstructure of Atlantic mackerel during storage observed by cryo-scanning electron microscopy (Cryo-SEM) are shown in Figure 7. The section perpendicular to the muscle fiber direction of the sample stored for 0 days showed many holes with a smooth surface in the muscle fiber, which was formed by the ice crystals during frozen storage. With the increase in drip loss of Atlantic mackerel during storage, the myofibrils contracted and fractured, and the holes became dense and irregular. Granular materials appeared on the surface of holes after three days of storage for both 4 °C and 0 °C. A similar phenomenon also appeared in bighead carp during storage, which was explained by the ruptures of the endomysium and the perimysium and the outflow of sarcoplasm [39]. The moisture reduction and protein denaturation of Atlantic mackerel after refrigerated storage could be explained by the microstructure changes.

### 3.5. Physicochemical Analysis

**Figure 8:**
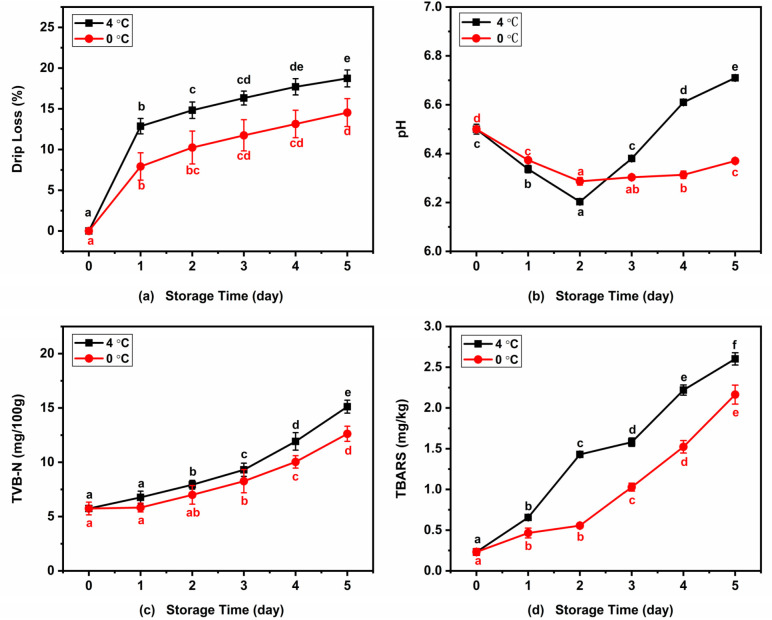
Variations of (**a**) drip loss, (**b**) pH, (**c**) TVB-N, and (**d**) TBARS of Atlantic mackerel during storage at 4 °C and 0 °C. Different letters a–f mean significant difference (*p* < 0.05).

Variations in the drip loss of Atlantic mackerel during storage at 4 °C and 0 °C are shown in Figure 8a. The drip loss of Atlantic mackerel significantly increased to 18.73% and 14.54% after five days of storage at 4 °C and 0 °C, respectively. This trend was in agreement with the changes in drip loss in sea bream during storage under different conditions [40]. Drip loss including water and lipid loss increased rapidly in the early storage stage and slightly in subsequent storage, which was consistent with the change in A_22_. The increase in drip loss of mackerel muscle during storage might be ascribed to the breakdown of protein, which led to the decrease of the WHC of muscle. Generally, lower storage temperature leads to lower drip loss.

Figure 8b shows the changes in the pH values of mackerel muscle with the increase in storage time under two refrigeration conditions. The pH values both declined significantly at the beginning of 4 °C and 0 °C storage, which reached the lowest values of 6.20 and 6.29 after two days of storage, respectively. With the further increase of storage time, the pH value of Atlantic mackerel samples stored at 4 °C increased significantly while that of the samples stored at 0 °C increased slightly. This was consistent with the result of the grass carp fillets during 0 °C storage, in which pH value decreased within the first three days and then increased during the following storage [41]. The pH value decrease during the first two days of storage was due to the degradation of adenosine-triphosphate (ATP) and creatine phosphate, and the subsequent increase was caused by the formation of alkaline substances related to protein degradation during refrigeration.

TVB-N mainly consists of amines such as methylamine, dimethylamine, and trimethylamine, which have been considered as important quality indicators of aquatic products. Figure 8c shows the changes in TVB-N values of Atlantic mackerel during refrigeration of 4 °C and 0 °C. Significant increases in TVB-N content from an value of 5.74 ± 0.56 mg/100 g to 15.12 ± 0.60 and 12.62 ± 0.70 mg/100 g were observed after five days of storage at 4 °C and 0 °C. The increase in TVB-N value in fish during cold storage is mainly due to the alkalinity substances produced by the protein degradation by enzymatic reactions and microbial activity [42].

The lipids in Atlantic mackerel are prone to oxidize during storage, and the degree of lipid oxidation can be evaluated by the TBARS value [7]. Figure 8d shows changes in the TBARS value of Atlantic mackerel during 4 °C and 0 °C storage. The initial TBARS value at the beginning of the storage was 0.23 ± 0.04 mg/kg, which increased to 2.60 ± 0.08 mg/kg and 2.16 ± 012 mg/kg after five days of storage at 4 °C and 0 °C, respectively. Wang et al. reported that the storage at 0 °C was more effective at inhibiting lipid oxidation of salmon than 4 °C [19]. Free radicals produced by the lipid oxidation can promote protein oxidation to form carbonyl groups and change the secondary structure. Increased lipid oxidation was associated with protein carbonyl group formation and β-turn and β-sheet changes during storage.

### 3.6. Color Analysis

Color is a common parameter to evaluate the quality of food. The effects of the 4 °C and 0 °C storage processes on the lightness (L*), redness (a*), and yellowness (b*) values of Atlantic mackerel are shown in Table 1. With the increase of refrigeration time, the L* values of samples stored at both 4 °C and 0 °C increased significantly, and a higher storage temperature led to higher L* value. The changes in heme proteins (particularly the heme-ring destruction) were considered to be responsible for the increased L* values during the frozen storage of herring fillet [43]. The increase of L* in superchilled salmon was also thought to be due to the white spots on the surface of fillets caused by drip channels [44]. The a* values were relatively stable, while the b* values increased significantly during storage. The lipid oxidation was considered to be the main reason for the increase in yellowness for fish, which was also supported by the increase in TBARS value in this study.

### 3.7. Correlation Analysis between Protein Oxidation, Physicochemical Qualities, and Water Status

Correlation coefficients between protein oxidation indicators, physicochemical qualities, and LF-NMR relaxation times are shown in Table 2. *T*_21_ showed significant correlations with the contents of carbonyl and total sulfhydryl group for the samples stored at 4 °C. *T*_22_ was positively correlated with the contents of myofibrillar protein and total sulfhydryl, while negatively correlated with the contents of sarcoplasmic protein, carbonyl, disulfide bond, and surface hydrophobicity as well as drip loss, TBARS, TVB-N, L*, and b* during 4 and 0 °C storage. During storage, changes in total sulfhydryl content, disulfide bond content, and hydrophobicity affect the water binding ability of proteins. Significant correlations between relaxation time of immobilized water and physicochemical indexes were also observed in tuna [20]. Pearson’s correlation analysis proved the excellent correlations between *T*_22_ and the protein oxidation indicators and physicochemical parameters, which indicated that the quality changes of Atlantic mackerel during refrigerated storage could be monitored by LF-NMR.

## 4. Conclusions

In this study, the moisture migration, protein oxidation, microstructure, and physicochemical qualities of Atlantic mackerel (*Scomber scombrus*) during storage at 4 and 0 °C were investigated. Three proton components assigned to bound water, immobilized water, and lipid were observed in mackerel by LF-NMR relaxation. With the prolongation of refrigeration time, the mobility and abundance of immobilized water decreased. MRI images also displayed the decrease in water content of mackerel during storage. The refrigerated storage also caused the oxidation and the change of the secondary structure of the mackerel protein, the fracture of myofibrils, and the changes in physicochemical parameters.

## Figures and Tables

**Table 1:** Variations of color parameters of Atlantic mackerel during refrigeration at 4 °C and 0 °C.

Storage Time (D)	L*		a*		b*	
	4 °C	0 °C	4 °C	0 °C	4 °C	0 °C
0	45.83 ± 1.51 ^a^	45.83 ± 1.51 ^a^	−1.67 ± 0.47 ^a^	−1.67 ± 0.47 ^ab^	7.50 ± 0.50 ^a^	7.50 ± 0.50 ^a^
1	48.71 ± 1.11 ^b^	47.70 ± 0.56 ^ab^	−1.79 ± 0.47 ^a^	−1.88 ± 0.55 ^ab^	8.78 ± 0.38 ^ab^	7.82 ± 1.25 ^a^
2	53.46 ± 1.27 ^c^	50.44 ± 3.18 ^bc^	−1.78 ± 0.19 ^a^	−2.02 ± 0.46 ^a^	10.88 ± 1.67 ^bc^	9.22 ± 0.58 ^a^
3	54.79 ± 1.13 ^cd^	51.81 ± 1.35 ^bc^	−1.37 ± 0.72 ^a^	−2.27 ± 0.26 ^a^	11.52 ± 2.09 ^cd^	9.86 ± 1.37 ^ab^
4	56.57 ± 1.17 ^de^	54.40 ± 3.99 ^c^	−2.10 ± 0.08 ^a^	−1.08 ± 0.35 ^b^	11.85 ± 0.29 ^cd^	11.93 ± 1.80 ^b^
5	57.50 ± 1.74 ^e^	54.87 ± 2.09 ^c^	−1.51 ± 0.56 ^a^	−2.48 ± 0.69 ^a^	13.70 ± 0.87 ^e^	12.21 ± 1.56 ^b^

Different letters ^a–e^ in the same column mean significant difference (*p* < 0.05).

**Table 2:** Correlation coefficients between sarcoplasmic protein content, myofibrillar protein content, CP content, total SH content, disulfide bond content, surface hydrophobicity, drip loss, pH, TBARS, TVB-N, L*, a* and b* with *T*_21_ and *T*_22_ obtained by Pearson’s correlation analysis.

Parameter	4 °C		0 °C	
	*T*_21_	*T*_22_	*T*_21_	*T*_22_
Sarcoplasmic protein content	−0.269	−0.706 **	−0.536 *	−0.899 **
Myofibrillar protein content	0.573 *	0.832 **	0.458	0.650 **
Total CP content	−0.638 **	−0.849 **	−0.446	−0.542 *
Total SH content	0.617 **	0.856 **	0.466	0.681 **
Disulfide bond content	−0.242	−0.597 *	−0.573 *	−0.892 **
Surface hydrophobicity	−0.323	−0.641 **	−0.430	−0.802 **
Drip loss	−0.496 *	−0.750 **	−0.492 *	−0.792 **
pH	−0.266	−0.292	0.348	0.536 *
TBARS	−0.427	−0.822 **	−0.533 *	−0.754 **
TVB-N	−0.402	−0.739 **	−0.535 *	−0.785 **
L*	−0.462	−0.750 **	−0.584 *	−0.721 **
a*	−0.203	−0.760 **	−0.198	0.142
b*	−0.588 *	−0.790 **	−0.494 *	−0.690 **

* *p* < 0.01 and ** *p* < 0.05.

## Data Availability

The data that support the findings of this study are available from the corresponding author upon reasonable request.

## Supplementary Materials

The following are available online at https://www.mdpi.com/2304-8158/10/2/214/s1, Figure S1: *T*_2_ curves of fresh and freeze-dried Atlantic mackerel.
