# Supplementary material for: Influence of Refrigerated Storage on Water Status, Protein Oxidation, Microstructure, and Physicochemical Qualities of Atlantic Mackerel (Scomber scombrus)
Source: Foods. 2021 Jan 21;10(2):214. doi: 10.3390/foods10020214 (PMC7909831; doi:10.3390/foods10020214)
Supplement: Supplementary file 1 [file foods-10-00214-s001.pdf]

Supplementary Materials

Influence of refrigerated storage on water status, protein oxidation, microstructure and physicochemical qualities of Atlantic mackerel (*Scomber scombrus*)

Rong Lin [1,2,3], Shasha Cheng [1,2,3,*], Siqi Wang [1,2,3] Mingqian Tan [1,2,3] and Beiwei Zhu [1,2,3]

[1] National Engineering Research Center of Seafood, School of Food Science and Technology, Dalian Polytechnic University, Dalian 116034, China; 18308320017021@xy.dlpu.edu.cn (R.L.); 17308320017012@xy.dlpu.edu.cn (S.Q.W.); mqtan@dlpu.edu.cn (M.Q.T.); zhubw@dlpu.edu.cn (B.W.Z.)

[2] Collaborative Innovation Center of Seafood Deep Processing, Dalian 116034, China

[3] National Engineering Research Center of Seafood, Dalian 116034, Liaoning, China

* Correspondence: chengss@dlpu.edu.cn; Tel.: +86-411-86318657

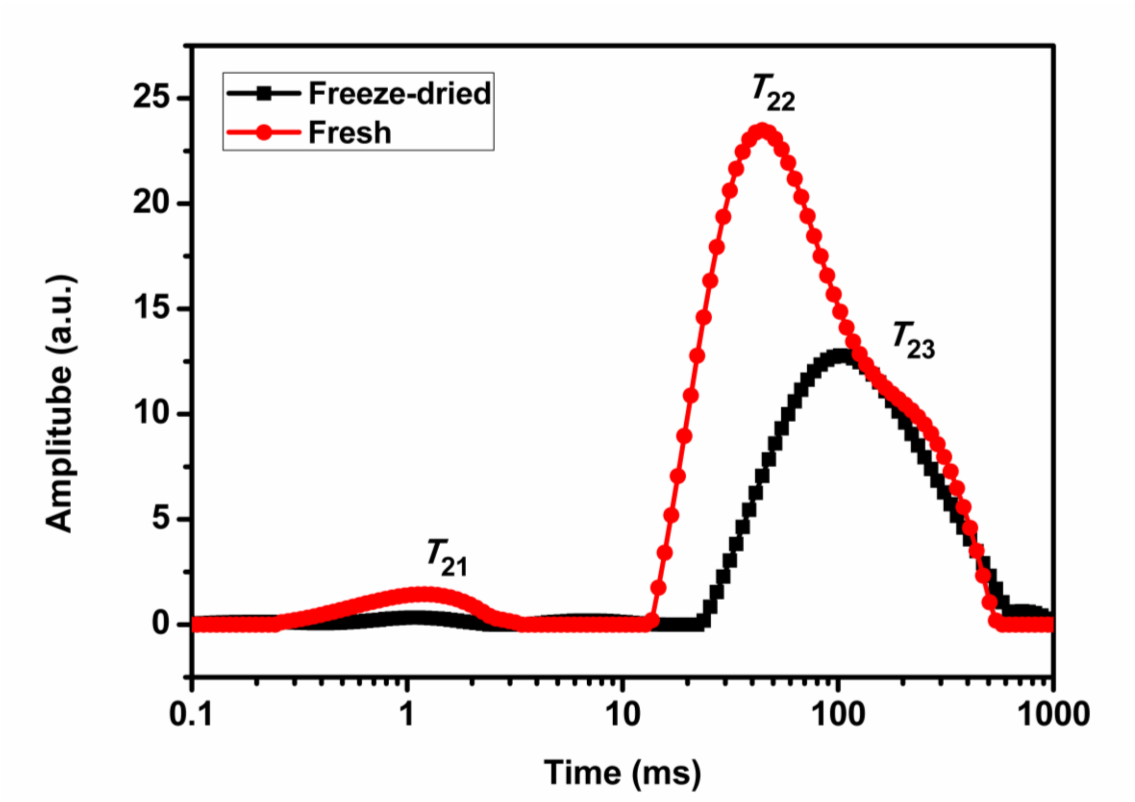

Figure S1. T_2 curves of fresh and freeze-dried Atlantic mackerel.
